# Editorial: Scanning Probe Microscopies and Related Methods in Biology

**DOI:** 10.3389/fmolb.2021.657939

**Published:** 2021-03-29

**Authors:** Andreas Engel, David Alsteens, Daniel J. Müller

**Affiliations:** ^1^ Department of Biozentrum, Faculty of Science, University of Basel, Basel, Switzerland; ^2^ Université catholique de Louvain, Louvain Institute of Biomolecular Science and Technology, Louvain-la-Neuve, Belgium; ^3^ Department of Biosystems Science and Engineering, ETH Zurich, Zurich, Switzerland

**Keywords:** scanning probe microscopy, atomic force microscopy, single molecule force spectroscopy, amyloid fibers, liposome mechanics, neurotransmitter/sodium symporter

Scanning Probe Microscopies and Related Methods in Biology is a timely research topic considering the wide range of their biological applications that have emerged in recent year. These methods measure and manipulate molecular interactions that dictate all processes in life at the single-molecule level. Progress in measuring and imaging such interactions keeps providing new insights into the complexity of life. The collection of contributions in this research topic give a view into the state-of-art of single molecule force spectroscopy and an insight into a diverse range of applications.

Readers have shown a significant interest in learning about progress in instrumentation for single molecule force spectroscopy Yang et al. as well as in practical aspects of its application Sumbul et al., 2020.

Liposomes become increasingly important not only in basic research but also as carriers of drugs or other agents such as m-RNA Reichmuth et al., 2016.

Therefore, Atomic Force Microscopy (AFM) has been used to characterize dimensions and mechanical of artificial liposomes and native vesicles over many years. An up-to-date protocol is a great help for an experimenter entering the field Vorselen et al.

Devastating neurodegenerative disorder as Alzheimer’s disease (AD) or Parkinson’s disease are major challenges of molecular medicine. Since the development of AD relates to the accumulation of amyloid β (Aβ) oligomers, understanding the molecular mechanisms defining the conversion of physiologically important monomers of Aβ proteins into neurotoxic oligomeric species is the key for the development of treatments and preventions of AD. Similarly, understanding of alpha-synuclein misfolding and neurotoxicity is important. AFM has been used to characterize assembly of amyloid fibers by time-lapse series before Goldsbury et al., 1999.

Two original research papers now demonstrate how beneficial the use of AFM for the characterization of amyloid β (Aβ) oligomers Maity and Lyubchenko; Feuillie et al.

In contrast to disease-inducing amyloid fiber formation, the fibronectin (FN) fibrillogenesis is an important process, e.g., in wound healing. It has also been analyzed by time-lapse atomic force microscopy (AFM). In the course of such experiments, light was observed to impair the capability of FN to form fibers. A first systematic study of this phenomenon is presented in Gudzenko and Franz.

Although crystal structures and modeling are the major tools in medicinal chemistry, molecular pharmacology experiments produced conflicting results on ligand binding sites in neurotransmitter/sodium symporters. A minireview on binding sites in the serotonin transporter sheds light on its allosterically linked binding sites. Again, single molecule force spectroscopy is the tool of choice to unravel the complexity of the transporter’s multiple binding sites Zhu et al.

In times of a pandemic catastrophe, the efficiency of anticontamination fluids is critical. High-resolution microscopies including AFM are excellent tools to assess the effect of different alcohol solutions by simple observations of the morphological changes of viral samples induced by the treatments. The experiments on Adenovirus Martín-González et al. could be adapted under safe conditions for assessing actions to eliminate Covid-19 contamination.

On-going developments of AFM technology bring new imaging modes offering the acquisition of multiple parameters of the addressed biological structure or unprecedented time resolution for monitoring protein dynamics. Also, multifunctional hollow cantilevers have been designed for delivery or extraction of biomolecules to/from single cells. This progress is not summarized here, but may become an interesting research topic in the future.
